# Draft Genome Sequence of Bacillus velezensis Strain Krd-20 with Antifungal Activity, a Biotechnologically Important Isolate from Wheat Root Zone

**DOI:** 10.1128/mra.00656-22

**Published:** 2022-09-20

**Authors:** Sergey V. Kopyltsov, Alexander V. Milovanov, Anastasia V. Elisiutikova, Darja S. Savenkova, Kristina O. Petrova, Dmitry V. Kwon, Andrej G. Koshchaev

**Affiliations:** a Department of Biotechnology, biochemistry and biophysics, Kuban State Agrarian University named after I. T. Trubilin, Krasnodar, Russia; b Laboratory of Plant and Animal Genetics, Kuban State Agrarian University named after I. T. Trubilin, Krasnodar, Russia; c Kurchatov Center for Genome Research, Moscow, Russia; SIPBS, University of Strathclyde

## Abstract

Bacillus velezensis Krd-20 strain with antifungal activity was isolated from the wheat rhizosphere. This strain is used to suppress fungi of the Fusarium sp. when growing oyster mushroom (Pleurotus ostreatus). Genome assembling resulted in 44 contigs with a total length of 3939663 bp were obtained, the GC content is 46.4%.

## ANNOUNCEMENT

Some strains of *Bacillus sp.* have antifungal properties against pathogenic fungi. They are valuable for agricultural enterprises in the fight against diseases of economically important crops (1, 2). Some species of the *Bacillus* sp. might also produce lipopeptides of three groups that inhibit the development of pathogenic microflora: iturin, surfactin, and fengycin (3).

During the isolation of native microflora from the soil of wheat rhizosphere (0 to 6 in. depth), *Bacillus* sp. strains with an antifungal effect were isolated (4). Field and sampling site located at the educational and experimental farm “Kuban” of the Kuban State Agrarian University (coord.: 45°04′02.97″N 38°52′56″E). Isolates were incubated on Potato Dextrose Agar (PDA) medium at 28°C for 48 h in the thermostat. Their properties were tested by coculturing strains of bacteria and fungi Fusarium oxysporum, and then zones of growth inhibition were evaluated (4). During the study morphological and tinctorial properties belonging to the genus *Bacillus sp.* were confirmed. Belonging to this genus was determined by the beige color of bacterial colonies, the rod-shaped similarity, the ability to stain with fuchsin and to sporulation.

Bacterial DNA was isolated using the Puregene Yeast/Bact. kit B (Qiagen, Germany). DNA concentration was measured by a Qubit fluorometer (ThermoFisher) implementation. Fragment selection was performed using magnetic particles from DNA Clean Beads (AMPure). Libraries were prepared using a DNA Prep, (M) Tagmentation kit (Illumina Inc, USA). Whole-genome sequencing was performed in NovaSeq 6000 (Illumina). As a result, 3,389,670 paired-end reads 151 bp long were obtained. Read quality control was performed with FastQC v.0.11.9 (5). Removal of technical sequences (adapters) was carried out using Trimmomatic v.0.39 (6). The genome was assembled using the UGENE program (7) with the built-in SPAdes v.3.15.3 algorithm (8) with default “auto” as k-mers number. The quality of genomic assemblies was evaluated with the QUAST v.5.0.2 package (9). Genome annotation was performed using the NCBI Prokaryotic Genome Annotation Pipeline (PGAP) v.5.3 (10). Default parameters were used for all software.

Paired-end reads were used to assemble Krd-20 genome, and 44 contigs with a total length of 3939663 bp were obtained. The *N*_50_ value was 286045, the GC content was 46.4%, and the genome coverage was 40.0×. The total number of protein-coding genes was 3866, RNAs −81. Out of 3866 CDS, 3797 genes (98.22%) were predicted to be protein-coding sequences.

The taxonomic position was determined using dDDH method (11) in Type (Strain) Genome Server (TYGS) (12). According to the TYGS results, the studied genome was identified as Bacillus velezensis strain. According to the TYGS results, the studied strain was most similar to the Bacillus velezensis NRRL B-41580 (GenBank number GCA_001461825.1).

The use of tblastn made it possible to predict sequences in the genome of the strain which may be involved in the biosynthesis of fungistatic lipopeptides, namely, iturin, fengycin, and surfactin. The amino acid sequences that already deposited at the NCBI, were used for the search (GenBank numbers: QDF52476.1; QDF56154.1; QDF52473.1; API81806.1; API81807.1; API81808.1; SNY69789.1; SNY69794.1; SMF34656.1).

### Data availability.

The genome was deposited in GenBank under the following accession numbers: BioSample SAMN25352221, BioProject PRJNA801484, SRA SRR19880915, and GenBank GCA_021892375.1.
